# Implementing Clinical Decision Support Tools and Pharmacovigilance to Reduce the Use of Potentially Harmful Medications and Health Care Costs in Adults With Heart Failure

**DOI:** 10.3389/fphar.2021.612941

**Published:** 2021-04-30

**Authors:** Armando Silva Almodóvar, Milap C. Nahata

**Affiliations:** ^1^ College of Pharmacy, The Ohio State University, Columbus, OH, United States; ^2^ SinfoniaRx: A TRHC Solution, Tucson, AZ, United States; ^3^ College of Medicine, The Ohio State University, Columbus, OH, United States

**Keywords:** heart failure, potentially harmful medication, health care utilization, clinical decision support systems, medication therapy management, pharmacovigilance

## Abstract

Heart failure (HF) is associated with significant morbidity, mortality, compromised quality of life and socioeconomic burden worldwide. This chronic condition is becoming an increasingly important concern given the increased prevalence of HF among aging populations. Significant contributors toward escalating health care costs are emergency room visits and hospitalizations associated with HF. An important strategy to improve health care outcomes and reduce unnecessary costs is to identify and reduce the prescribing of potentially harmful medications (PHMs) among adults with HF. Previous studies in patients with HF found roughly 10–50% of them were prescribed at least one PHM in ambulatory care and inpatient health care settings. This opinion highlights recent findings from studies assessing prevalence of PHMs, associations between PHM prescribing and characteristics, and what can be done to improve patient outcomes and reduce the use of PHMs and associated health care costs in adults with HF.

## Introduction

Heart failure (HF) is associated with compromised quality of life, and significant morbidity, mortality, and socioeconomic burden worldwide. It is estimated that up to 7% of the population in some industrialized countries is diagnosed with HF (Savarese and Lund, 2017). A systematic review reported annual per patient cost of care ranged from $868 to $25,532 depending on the country (Lesyuk et al., 2018). Inpatient treatment of heart failure is estimated to comprise 44–96% of the overall cost of treatment (Lesyuk et al., 2018). Two-thirds of patients with HF experienced a rehospitalization within the first year hospital discharge in the US (Curtis et al., 2008). Reducing avoidable exacerbations of HF and optimizing medication regimens are necessary to mitigate avoidable health care utilization.

It is estimated that patients with HF utilize 7 prescriptions daily in addition to over-the-counter medications and supplements (Masoudi et al., 2005). Furthermore, one in two patients following a HF-related hospitalization utilized more than 10 prescriptions chronically (Unlu et al., 2019). It is estimated that 82% of patients using more than 7 medications may experience a significant drug-drug interaction (Goldberg et al., 1996). Additionally, medication related treatment failure and new medical problems may cost the US approximately $528.4 billion annually (Watanabe et al., 2018). Medication related problems such as drug-drug interactions, drug-disease interactions, and suboptimal dosing of medications can occur in fragmented health care systems where patients utilize multiple health care providers. This is problematic among patients with HF in whom worsening disease control can quickly lead to avoidable emergency department (ED) visits and hospitalizations.

The American Heart Association (AHA) and the European Society of Cardiology (ESC) published statements detailing a list of potentially harmful medications (PHMs) known to exacerbate or cause HF and included a detailed description of their quality of evidence (Page et al., 2016; El Hadidi et al., 2020). Additionally, heart failure management guidelines published by the AHA in collaboration with the American College of Cardiology and the Heart Failure Society of America (Yancy et al., 2017), the ESC (Ponikowski et al., 2016), the National Heart Foundation of Australia and Cardiac Society of Australia and New Zealand (Atherton et al., 2018), and the Japanese Circulation Society in collaboration with the Japanese Heart Failure Society (Tsutsui et al., 2019) identified certain medications that should be avoided among patients with HF. Despite these publications, little has been done to reduce prescribing of these medications among patients with HF through a systematic approach. Recent research has demonstrated that dedicated clinical decision support systems (CDSS) can improve guideline directed prescribing for the treatment of HF in ambulatory care settings (McKie et al., 2020) and electronic engagement of patients can also positively improve prescribing (Allen et al., 2021). However, a review by Kao et al. (Kao et al., 2020) reported on the need for greater innovation within electronic health records (EHRs), such as triggered alerts within a CDSS to reduce prescribing of PHMs for optimal management of patients with HF. This opinion reviewed evidence describing the prevalence of PHM prescribing among patients with HF and has suggested strategies about how health care systems can reduce prescribing of PHMs through triggered alerts within a CDSS and pharmacovigilance programs to reduce medication burden, and potentially avoidable health care utilization.

## Potentially Harmful Medication Prescribing

Since the publication of the AHA scientific statement, several studies have examined the prescribing of PHM among adults. Presently, the analysis of PHM prescribing among patients with HF can be divided into three settings: hospitalizations (Caughey et al., 2019; Alvarez et al., 2020; Goyal et al., 2020) administrative claims, (Alvarez et al., 2019; Silva Almodóvar and Nahata, 2020) and within an outpatient clinic (Brinker et al., 2020). These studies in large part assessed PHMs among patients in the United States (US) (Alvarez et al., 2019; Alvarez et al., 2020; Brinker et al., 2020; Goyal et al., 2020; Silva Almodóvar and Nahata, 2020) while one study assessed PHM prescribing in Australia (Caughey et al., 2019). Complete information on the studies assessed in this opinion can be found in Table 1.

**TABLE 1:** Summary of studies reporting potentially harmful medication prescribing in patients with heart failure.

Study Authors (publication year)	Setting (study year/s)	Inclusion Criteria	Exclusion Criteria	Rule set utilized and applied in the study	Number of patients assessed (count, % with PHM)	Characteristics associated with PHM^a^ (OR, 95% CI)	Most common medications reported as PHM^b^ (n, %)
Goyal et al. (2020)	Inpatient Hospitalization during REGARDS study enrollment in the US (2003–2014)	Medicare Part A enrollment for 90 days following hospitalization, ≥65 years of age, participant of REGARDS study, hospitalized for HF	Hospice referral at discharge from hospital, without medication data at hospital admission or discharge	2016 AHA Scientific Statement Drugs That May Cause or Exacerbate heart failure (medications limited to those as having potentially life-threatening effects that could lead to a hospitalization or emergencydepartment visit)	558 (228, 41%)	Logistic regression assessing association with PHM prescribing after discharge: diabetes (1.80, 1.18–2.75) small hospital size (1.93, 1.18–3.16)	At hospital admission: Albuterol (92, 16%) Metformin (55, 10%) NSAIDS (50, 9%) Diltiazem (39, 7%) Thiazolidinediones (35, 6%) At hospital discharge: Albuterol (123, 22%) Metformin (41, 7%) NSAIDS (18, 3%) Diltiazem (42, 8%) Thiazolidinediones (20, 4%)
Alvarez et al. (2020)	90 days post discharge from hospitalization identified from CMS data files of a nationally representative 5% Medicare sample in the US (2013–2016)	Medicare enrollment, ≥66 years of age, HF discharge between April 2014–September 2016, with primary diagnosis of HFrEF, enrolled in Medicare Part D at hospital discharge, filled a prescription for an ACEi, ARB, or ARNi, and an HF-specific beta-blocker (metoprolol succinate, bisoprolol, or carvedilol) within 90 days from discharge	Not enrolled in Medicare Part D, diagnosis of metastatic cancer or malignant tumor, ESRD, death during the index hospitalization, not discharged home or left hospital against medical advice	2013 ACC/AHA HF guidelines: NSAIDs (diclofenac, ibuprofen, naproxen, meloxicam, indomethacin, celecoxib, ketorolac, etodolac, nabumetone, diflunisal, fenoprofen, flurbiprofen, ketoprofen, mefenamic oxaprozin, piroxicam, tolmetin), thiazolidinediones (pioglitazone and rosiglitazone), antiarrhythmics (flecainide, dronedarone), and non-dihydropyridine CCBs (diltiazem, verapamil)	90 days post discharge 8993 (1077, 12%) 365 days post discharge (1721, 19.14%)	Multivariate regression assessing association with PHM prescribing after discharge: Female (1.25, 1.08–1.46) Hispanic (1.49, 1.18–1.88) Severe Obesity (1.38, 1.10–1.74) Atrial Fibrillation (1.37, 1.18–1.59) Diabetes (1.37, 1.18–1.59) Chronic Lung Disease (1.44, 1.24–1.68) Pre-hospitalization PHD Exposure (14.99, 12.94–17.36) Ischemic heart disease (0.77, 0.66–0.90) Implantable Cardioverter Defibrillator (0.80, 0.63–0.999) Renal Failure (0.78, 0.67–0.93)	90 days post discharge NSAIDs (610, 6.7%) CCBs (426, 474%) 365 days post discharge NSAIDs (1185, 13.18%) CCBs (525, 5.84%)
Caughey et al. (2019)	Administrative health claims from Australian Government Department of Veteran Affairs (DVA) (2012)	Hospitalized with HF, eligible for all health services subsidized by the DVA in the 12 months before the start date of the study	Not reported	2016 AHA Scientific Statement Drugs That May Cause or Exacerbate Heart Failure, 2011 update to National Heart Foundation of Australia and Cardiac Society of Australia and New Zealand Guidelines for the prevention, detection, and management of chronic heart failure in Australia, 2006 (omitted anesthesia medicines and dronedarone due to its unavailability in the dataset or country respectively)	4069 (2435, 59.8%)	Not reported	120 days prior to hospitalization Albuterol (832, 20.4%) Systemic corticosteroids (709, 17.4%) Tricyclic Antidepressants (380, 9.3%) Metformin (338, 8.3%) Tamsulosin (151, 7.3%) Non-selective COX Inhibitors (251, 6.2%) Topical B-Blockers (232, 5.7%) Diltiazem (210, 5.2%) 120 days after hospitalization Albuterol (832, 20.4%) Systemic corticosteroids (709, 17.4%) Tricyclic Antidepressants (380, 9.3%) Metformin (338, 8.3%) Tamsulosin (151, 7.3%) Non-selective COX Inhibitors (251, 6.2%) Topical B-Blockers (232, 5.7%) Diltiazem (210, 5.2%)
Alvarez et al. (2019)	Outpatient pharmacy claims from Truvan Health Market Scan Claims database in the US (2011–2015)	Diagnosed with systolic HF, between 18–65 years of age	COPD on steroids, ESRD, malignant neoplasm with/without metastatic disease, with less than 6 months of claims from enrollment date, no pharmacy coverage	2016 AHA Scientific Statement Drugs That May Cause or Exacerbate Heart Failure (oral medications with A or B level evidence with major potential for induction or precipitation of HF)	40,966 (9954, 24.3%)	Logistic regression assessing association with PHM prescribing: Female sex (1.16, 1.10–1.22) Osteoarthritis (1.70, 1.61–1.79) Hypertension (1.36, 1.25–1.47) Diabetes mellitus (1.52, 1.44–1.59) Atrial fibrillation (1.23, 1.17–1.29) Myocardial infarction (0.76, 0.72–0.80) Neurological and/or psychiatric Disorders (1.42, 1.35–1.50) Outpatient cardiology visit (1.74, 1.65–1.84) Polypharmacy (1.69, 1.59–1.79)	After HF diagnosis: NSAIDS (6710, 44%) Citalopram (1680, 11%) Diltiazem (1675, 11%) Sitagliptin (1438, 9.4%) Antiarrhythmics (1258, 8.3%)
Silva Almodóvar and Nahata (2020)	Outpatient pharmacy claims from Medicare insurance plan in the US (2018)	Medicare enrolled, MTM eligible, diagnosed with HF	Without evidence of prescription claims, only with a diagnosis code for HFpEF	2016 AHA Scientific Statement Drugs That May Cause or Exacerbate Heart Failure (oral or injectable medications with A or B level evidence with major potential for induction or precipitation of HF)	13,250 (7017, 53%)	Number of unique medications (1.05, 1.04–1.06) Female Sex (1.24, 1.15–1.33) Living in an area where more than 10% of individuals lived below the federal poverty line (1.25–1.36)	During study period: NSAIDs (3357, 25%) DPP4i (3117, 24%) Non-dihydropyridine CCBs (936, 7%)
Brinker et al. (2020)	Frankel Cardiovascular Center Heart Failure with Preserved Ejection Fraction Clinic in the US (2016–2019)	Participation in clinic	Not reported	2016 AHA Scientific Statement Drugs That May Cause or Exacerbate Heart Failure: medications that posed a major risk of causing or exacerbating HF	231 (119, 52%)	Not reported	During study period: Metformin (43.19%) Nondihydropyridine CCB (26, 11%) Citalopram or escitalopram (18.8%) Sulfonylurea (16.7%) NSAIDs (16.7%) Hydroxychloroquine (13.6%)

^a^ Only statistically significant associations were included.

^b^ Medications included with greater than 5% prevalence.

ACC, American college of cardiology; ACEi, angiotensin converting enzyme inhibitor; AHA, American heart association; ARB, angiotensin receptor blocker; ARNi, angiotensin receptor–neprilysin inhibitor; CI, confidence interval; CCB, calcium channel blockers; CMS, centers for medicare and medicaid services; COPD, chronic obstructive pulmonary disease; DPP4i, dipeptidyl peptidase-4 inhibitor; ESRD, end stage renal disease; HF, heart failure; HFpEF, heart failure preserved ejection fraction; HFrEF, heart failure reduced ejection fraction; ICD, international classification of diseases; MTM, medication therapy management; NSAIDS, nonsteroidal anti-inflammatory drugs; OR, odds ratio; PHM, potentially harmful medication; US, united states.

Goyal et al. (Goyal et al., 2020) assessed the prescribing of PHMs among older adults participating in a nationally representative cohort before and after a HF-related hospitalization. This study found 41% of individuals hospitalized for HF were using a PHM (Goyal et al., 2020). Upon discharge, 36% of patients still utilized a PHM. Alvarez et al. (Alvarez et al., 2020) conducted a similar analysis among Medicare patients with reduced ejection fraction HF (HFrEF) at 90 and 365 days after discharge from a HF-related hospitalization. This study found 12% of patients were with PHM at 90 days after a HF-related hospitalization and the prevalence increased to 19% at 1 year (Alvarez et al., 2020). It is important to note this study limited their identification of PHMs to medications mentioned in the 2013 ACC/AHA HF guideline potentially resulting in an underestimation of PHM prescribing (Alvarez et al., 2020).

Caughey et al. (Caughey et al., 2019) assessed PHM prescribing at 120 days before and after a HF-related hospitalization utilizing an Australian Department of Veteran Affairs claims database. Authors found almost 60% of their cohort were prescribed PHMs at 120 days prior to a HF-related hospitalization while 56% continued to be prescribed a PHM after 120 days (Caughey et al., 2019). The most common medications identified as PHMs in these studies included albuterol, metformin, non-dihydropyridine calcium channel blockers (CCBs), tricyclic antidepressants, systemic corticosteroids, and tamsulosin (Caughey et al., 2019; Alvarez et al., 2020; Goyal et al., 2020). These medications have been associated with an increased risk of hospitalization, increased (ED) visits, or exacerbation or precipitation of HF (Page et al., 2016).

Two studies assessed PHM prescribing by analyzing administrative claims data (Alvarez et al., 2019; Silva Almodóvar and Nahata, 2020). Alvarez et al. (Alvarez et al., 2019) found 24% of adults under 65 years of age with HFrEF were prescribed a PHM (Alvarez et al., 2019). A separate study using claims for one Medicare insurance plan assessed patients with HF who were enrolled in Medicare and eligible for Medication Therapy Management (MTM) services (Silva Almodóvar and Nahata, 2020). This study assessed medication prescribing in a Medicare cohort with significant comorbidity burden which was at greater risk for drug-drug and drug-disease interactions (Silva Almodóvar and Nahata, 2020). This study found 53% of patients were prescribed a PHM (Silva Almodóvar and Nahata, 2020). These studies may have underestimated the prevalence of PHM in their populations given they limited their PHMs to only medications with evidence derived from randomized clinical trials, meta-analyses, single randomized trials, or nonrandomized studies (Alvarez et al., 2019; Silva Almodóvar and Nahata, 2020). The most common PHMs prescribed in these studies were NSAIDs, nondihydropyridine CCBs, dipeptidyl peptidase-4 inhibitors (DPP4i), citalopram, specific antiarrhythmics, and thiazolidinediones (Alvarez et al., 2019; Silva Almodóvar and Nahata, 2020). In addition to the previously mentioned medications, use of NSAIDs, DPP4is, citalopram, antiarrhythmics, and thiazolidinediones among patients with HF may lead to a potentially avoidable hospitalization or ED visit (Page et al., 2016).

Finally, a study by Brinker et al. (Brinker et al., 2020) examined the prescribing of PHMs among a cohort with preserved ejection fraction HF (HFpEF) in one outpatient clinic. Approximately, 52% of patients were with a PHM. The most commonly prescribed PHMs in this study were metformin, non-dihydropyridine CCBs, and citalopram or escitalopram.

## Characteristics Associated With PHM Prescribing

Examining patient characteristics associated with the prescribing of PHM may inform which patient populations would likely benefit most from targeted interventions. Goyal et al. (Goyal et al., 2020) reported patients with diabetes and those admitted to small hospitals with PHM prescribing had greater odds of having a PHM at discharge. Among Medicare patients that were hospitalized with HF, patients with PHM prescribing prior to the hospitalization, female sex, atrial fibrillation, severe obesity, diabetes, and chronic lung disease were with higher odds of PHM after a HF-related discharge; patients with ischemic heart disease, implantable cardioverter defibrillator, and renal failure were with significantly lower odds of PHM prescribing after a HF-related discharge (Alvarez et al., 2020).

In adults under 65 years of age, polypharmacy, use of loop diuretics, an outpatient cardiology visit, female sex, and diagnoses of osteoarthritis, hypertension, diabetes mellitus, atrial fibrillation, peripheral vascular disorder, neurological/psychiatric disorders, and chronic obstructive pulmonary disease were associated with prescribing of PHM; patients with history of a myocardial infarction were with lower odds of PHM prescribing (Alvarez et al., 2019). Among Medicare patients who were eligible for an MTM service, female sex, increasing number of prescriptions, residence in higher levels of poverty and greater number of prescribers and pharmacies were associated with PHM prescribing (Silva Almodóvar and Nahata, 2020). It is important to note the only characteristic of prescribers assessed was prescriber specialty. One study found that physician primary care providers prescribed the largest number of PHMs among MTM eligible patients with HF (Silva Almodóvar and Nahata, 2020).

## Discussion

Numerous studies have provided clear evidence for the prescribing of PHMs among patients with HF, which can lead to unnecessary health care utilization. Inpatient care represented 44–96% of the global cost of the management of HF; it is estimated to represent approximately 62–84% of the annual costs in the US. (Lesyuk et al., 2018) An obvious question is: what can be done to address this issue? One strategy would be to implement triggered alerts in an electronic health record’s CDSS using rule sets adapted from the AHA scientific statement, ESC’s position statement and heart failure prescribing guidelines to identify and prevent prescribing of PHMs in patients with HF. Smaller hospitals relative to larger hospitals may benefit more from this type of intervention given patients at these hospitals had greater odds of having a PHM (Goyal et al., 2020).

This type of triggered alert within a CDSS can draw the health care provider’s attention to the patient’s previously established diagnosis of HF, the offending drug’s potential for harm, and suggest a safer medication. This would allow the provider to make the most educated therapeutic decision at the point of prescribing. To the authors’ knowledge current electronic interventions are primarily focused on improving prescribing of medications meant to treat HF and research is needed to evaluate the use of these technologies to reduce rates of PHM prescribing. A previous systematic review found implementation of CDSS improved provider compliance with clinical practice related to the screening and treatment of cardiovascular related illnesses (Njie et al., 2015). McKie et al. (McKie et al., 2020) found CDSS significantly improved guideline recommended treatment of patients with HF in a primary care setting. However, another study in patients with HF in a hospital setting reported roughly 3.6 alerts per patient resulting in provider alert fatigue (Wadhwa et al., 2008). The risk for alert fatigue emphasizes the need to carefully design the triggered alerts with user feedback to ensure optimal uptake and efficacy.

In addition to the implementation of well-designed triggered alerts, dedicated pharmacovigilance programs need to be implemented to identify and resolve potential drug-drug interactions, drug-disease interactions, and adverse events. Targeted programs for PHMs among patients with heart failure can reduce prescribing of PHMs and thus reduce potentially avoidable health care utilization. As an example, the Centers for Medicare and Medicaid Services currently requires Medicare insurance plan providers to utilize MTM programs to optimize health outcomes and reduce the risk of medication related adverse events (Medication therapy management Centers for Medicare and Medicaid Services, 2020). MTM programs may incorporate automated algorithm driven electronic reviews and manual reviews of medication claims by health care providers to decrease and prevent prescribing of harmful medications.

It would be important for these programs to utilize health care providers with expertize in the comprehensive management of multiple concurrent medications as patients with heart failure and comorbidities such as diabetes, severe obesity, hypertension, atrial fibrillation, chronic lung disease, osteoarthritis, hypertension, peripheral vascular disorder, or neurological/psychiatric disorders had greater odds of using a PHM (Alvarez et al., 2019; Alvarez et al., 2020; Goyal et al., 2020). Presently, MTM services are largely provided by clinical pharmacists who evaluate medication regimens and communicate with patients and prescribers to improve medication use (Centers for Medicare and Medicaid Services, 2018). These programs can address medication use after prescribing, given they would have access to diagnostic and prescription claims data for patients that may have been siloed across different health care systems and pharmacies. These features are especially important as patients with multiple prescribers and with multiple pharmacies presented with greater odds of having a PHM (Silva Almodóvar and Nahata, 2020).

Wide adoption of these programs across health care systems and insurance plans can significantly improve their ability to reduce the prevalence of PHMs. Previous research found MTM programs to be especially helpful in improving medication adherence and prescribing of medications in patients with HF (Perloth et al., 2013). However, the effects of MTM programs on reducing contraindicated medications among patients with HF may depend on the type of insurance program (Buhl et al., 2017). Targeted reviews within these programs have been found effective in initiating a large number of medication changes to reduce adverse outcomes (Buhl et al., 2017; Ferries et al., 2019). Thus, implementation of targeted programs and adoption of MTM services among health care systems and payers such as insurance plans may reduce prevalence of PHMs, hospitalizations, and health care utilization among patients with HF.

The prevalence of HF is expected to increase by 46% by 2030 (Benjamin et al., 2017). Given 10–50% of patients with HF utilized at least one PHM, there is an urgent need to develop and implement efficient and effective tools and programs to optimize medication management of patients with HF (Alvarez et al., 2019; Caughey et al., 2019; Alvarez et al., 2020; Brinker et al., 2020; Goyal et al., 2020; Silva Almodóvar and Nahata, 2020). The implementation of triggered alerts targeting PHM medications among patients with HF within the CDSSs across all health care systems and pharmacovigilance programs including MTM among insurance plan providers are likely to reduce the prescribing of PHMs, and thus improve health outcomes and reduce unnecessary health care utilization among adults with HF.
